# Supplementary material for: Structural Evaluation of a Nitroreductase Engineered for Improved Activation of the 5-Nitroimidazole PET Probe SN33623
Source: Int J Mol Sci. 2024 Jun 15;25(12):6593. doi: 10.3390/ijms25126593 (PMC11203732; doi:10.3390/ijms25126593)

Supplementary File S1: Supplementary Figures

Figure S1: Reaction scheme for flavoenzyme-mediated reduction of nitroaromatic substrates. The first step is the reductive half reaction, consisting of hydride transfer from NADH or NADPH to the bound FMN of a nitroreductase enzyme (NTR), and the oxidative half reaction consists of subsequent hydride transfer from the reduced FMN to the nitro group (-NO_2) on an aromatic ring (Ar) to form a nitroso species (-NO). A subsequent series of hydride transfers may then reduce the nitroso species to a hydroxylamine (-NHOH).

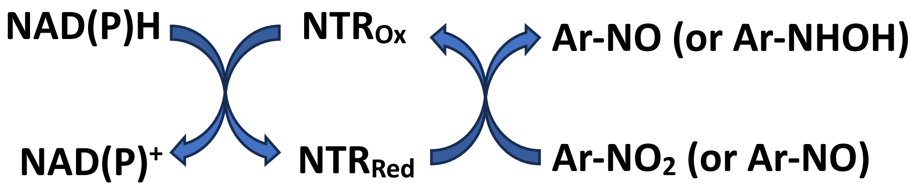

Figure S2: Active site overlay of AlphaFold-predicted and X-ray crystallography-determined structures. AlphaFold2 was used to predict the dimeric structure of *E. coli* NfsB F70A/F108Y. This predicted model was aligned with our X-ray crystallography determined structure (PDB code 8V5B) and the wild-type *E. coli* NfsB published structure (PDB code 1DS7). Key active site residues in the predicted model, 8V5B and 1DS7 are shown as green, pink or red sticks, respectively. The FMN cofactor is shown as yellow sticks. The surface represents the AlphaFold model. Alignments were generated for both active sites of the AlphaFold dimeric model. The overall RMSD was 0.342 Å on alignment of dimers.

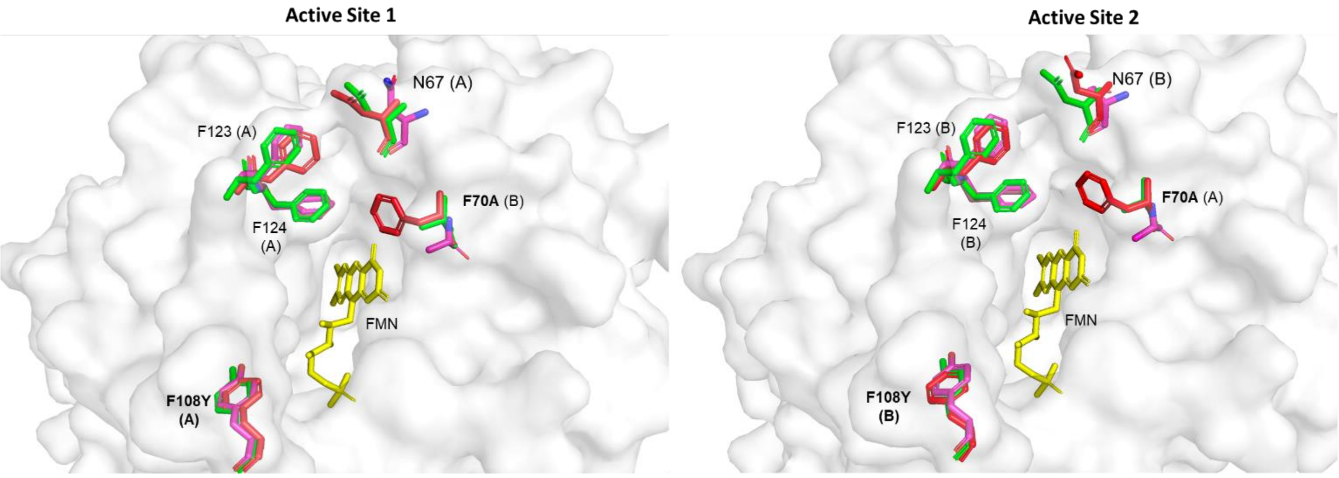

Supplement: Supplementary file 1 [file ijms-25-06593-s001.zip › ijms-3051797-figures.pdf]
