# Supplementary material for: Structural Evaluation of a Nitroreductase Engineered for Improved Activation of the 5-Nitroimidazole PET Probe SN33623
Source: Int J Mol Sci. 2024 Jun 15;25(12):6593. doi: 10.3390/ijms25126593 (PMC11203732; doi:10.3390/ijms25126593)
Supplement: Supplementary file 1 [file ijms-25-06593-s001.zip › ijms-3051797-supplementary.pdf]

Full wwPDB X-ray Structure Validation Report (i)

Dec 1, 2023 – 11:49 AM EST

PDB ID	:	8V5B
Title	:	Structure of the oxygen-insensitive NAD(P)H-dependent nitroreductase NfsB_Ec F70A/F108Y in complex with FMN
Deposited on	:	2023-11-30
Resolution	:	1.98 Å(reported)

This wwPDB validation report is for manuscript review

For Manuscript Review

This is a Full wwPDB X-ray Structure Validation Report.

This report is produced by the wwPDB biocuration pipeline after annotation of the structure.

We welcome your comments at *validation@mail.wwpdb.org*
A user guide is available at
https://www.wwpdb.org/validation/2017/XrayValidationReportHelp
with specific help available everywhere you see the (i) symbol.

The types of validation reports are described at
http://www.wwpdb.org/validation/2017/FAQs#types.

The following versions of software and data (see references (i)) were used in the production of this report:

MolProbity	:	4.02b-467
Mogul	:	1.8.5 (274361), CSD as541be (2020)
Xtriage (Phenix)	:	1.13
EDS	:	2.36
buster-report	:	1.1.7 (2018)
Percentile statistics	:	20191225.v01 (using entries in the PDB archive December 25th 2019)
Refmac	:	5.8.0158
CCP4	:	7.0.044 (Gargrove)
Ideal geometry (proteins)	:	Engh & Huber (2001)

1 Overall quality at a glance (i)

The following experimental techniques were used to determine the structure:
X-RAY DIFFRACTION

The reported resolution of this entry is 1.98 Å.

Percentile scores (ranging between 0-100) for global validation metrics of the entry are shown in the following graphic. The table shows the number of entries on which the scores are based.

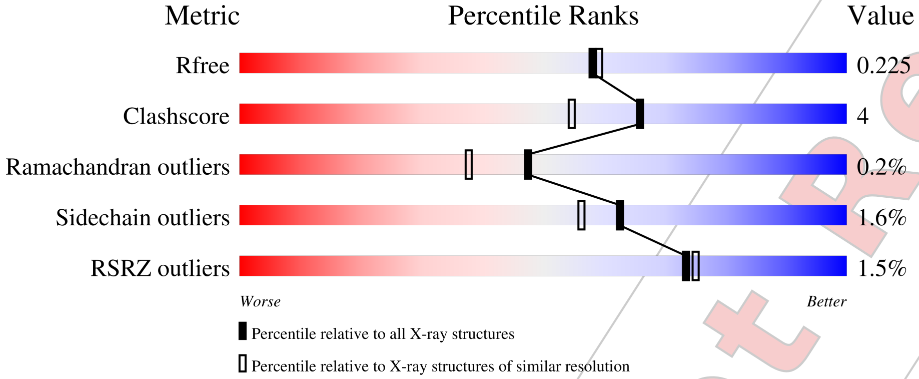

Metric	Whole archive (#Entries)	Similar resolution (#Entries, resolution range(Å))
R_{free}	130704	11647 (2.00-1.96)
Clashscore	141614	1014 (1.98-1.98)
Ramachandran outliers	138981	1006 (1.98-1.98)
Sidechain outliers	138945	1006 (1.98-1.98)
RSRZ outliers	127900	11410 (2.00-1.96)

The table below summarises the geometric issues observed across the polymeric chains and their fit to the electron density. The red, orange, yellow and green segments of the lower bar indicate the fraction of residues that contain outliers for >=3, 2, 1 and 0 types of geometric quality criteria respectively. A grey segment represents the fraction of residues that are not modelled. The numeric value for each fraction is indicated below the corresponding segment, with a dot representing fractions <=5% The upper red bar (where present) indicates the fraction of residues that have poor fit to the electron density. The numeric value is given above the bar.

Mol	Chain	Length	Quality of chain
1	A	217	93% 7%
1	B	217	2% / 96% •
1	C	217	% / 93% 7%

Continued on next page...

Ideal geometry (DNA, RNA) : Parkinson et al. (1996)
Validation Pipeline (wwPDB-VP) : 2.36

For Manuscript Review

Continued from previous page...

Mol	Chain	Length	Quality of chain
1	D	217	2% 91% 9%

The following table lists non-polymeric compounds, carbohydrate monomers and non-standard residues in protein, DNA, RNA chains that are outliers for geometric or electron-density-fit criteria:

Mol	Type	Chain	Res	Chirality	Geometry	Clashes	Electron density
3	ACT	C	304	-	-	X	-
3	ACT	D	302	-	-	X	-
3	ACT	D	303	-	-	X	-

For Manuscript Review

2 Entry composition (i)

There are 6 unique types of molecules in this entry. The entry contains 7332 atoms, of which 0 are hydrogens and 0 are deuteriums.

In the tables below, the ZeroOcc column contains the number of atoms modelled with zero occupancy, the AltConf column contains the number of residues with at least one atom in alternate conformation and the Trace column contains the number of residues modelled with at most 2 atoms.

- Molecule 1 is a protein called Dihydropteridine reductase.

Mol	Chain	Residues	Atoms					ZeroOcc	AltConf	Trace
1	A	217	Total 1665	C 1055	N 286	O 318	S 6	0	0	0
1	B	217	Total 1668	C 1057	N 286	O 319	S 6	0	0	0
1	C	217	Total 1663	C 1053	N 286	O 318	S 6	0	0	0
1	D	217	Total 1656	C 1047	N 285	O 318	S 6	0	0	0

There are 8 discrepancies between the modelled and reference sequences:

Chain	Residue	Modelled	Actual	Comment	Reference
A	70	ALA	PHE	engineered mutation	UNP A0A094VLP5
A	108	TYR	PHE	engineered mutation	UNP A0A094VLP5
B	70	ALA	PHE	engineered mutation	UNP A0A094VLP5
B	108	TYR	PHE	engineered mutation	UNP A0A094VLP5
C	70	ALA	PHE	engineered mutation	UNP A0A094VLP5
C	108	TYR	PHE	engineered mutation	UNP A0A094VLP5
D	70	ALA	PHE	engineered mutation	UNP A0A094VLP5
D	108	TYR	PHE	engineered mutation	UNP A0A094VLP5

- Molecule 2 is FLAVIN MONONUCLEOTIDE (three-letter code: FMN) (formula: $C_{17}H_{21}N_4O_9P$) (labeled as "Ligand of Interest" by depositor).

For Manuscript Review

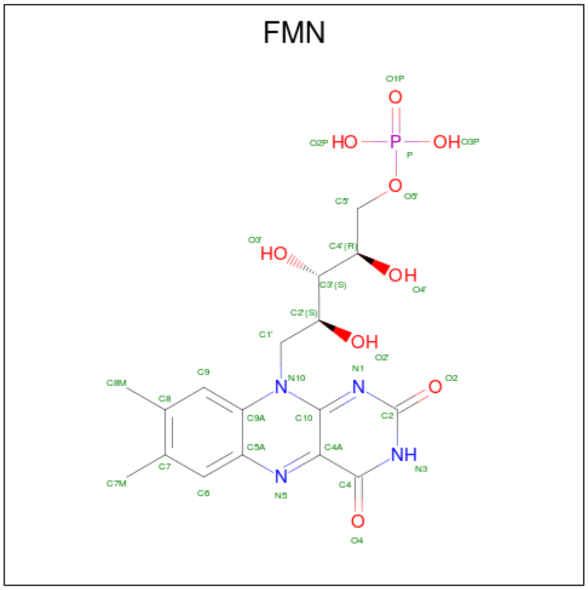

Mol	Chain	Residues	Atoms	ZeroOcc	AltConf
2	A	1	Total C N O P<br>31 17 4 9 1	0	0
2	B	1	Total C N O P<br>31 17 4 9 1	0	0
2	C	1	Total C N O P<br>31 17 4 9 1	0	0
2	D	1	Total C N O P<br>31 17 4 9 1	0	0

- Molecule 3 is ACETATE ION (three-letter code: ACT) (formula: $C_2H_3O_2$).

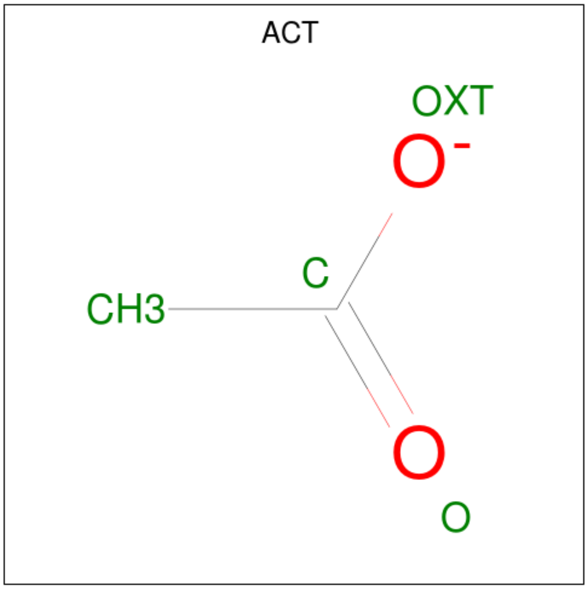

For Manuscript Review

Mol	Chain	Residues	Atoms	ZeroOcc	AltConf
3	A	1	Total C O<br>4 2 2	0	0
3	B	1	Total C O<br>4 2 2	0	0
3	C	1	Total C O<br>4 2 2	0	0
3	C	1	Total C O<br>4 2 2	0	0
3	C	1	Total C O<br>4 2 2	0	0
3	C	1	Total C O<br>4 2 2	0	0
3	D	1	Total C O<br>4 2 2	0	0
3	D	1	Total C O<br>4 2 2	0	0

- Molecule 4 is polyethylene glycol (three-letter code: P4K) (formula: $C_{30}H_{62}O_{15}$).

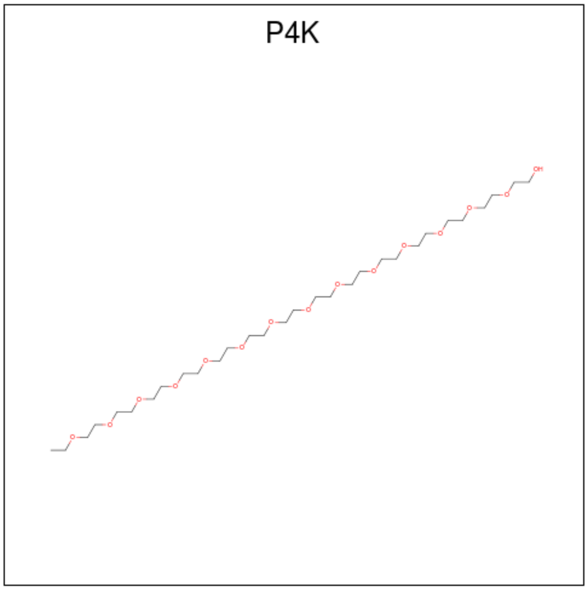

Mol	Chain	Residues	Atoms	ZeroOcc	AltConf
4	C	1	Total C O<br>7 4 3	0	0

- Molecule 5 is CHLORIDE ION (three-letter code: CL) (formula: Cl).

Mol	Chain	Residues	Atoms	ZeroOcc	AltConf
5	D	1	Total Cl<br>1 1	0	0

For Manuscript Review

- Molecule 6 is water.

Mol	Chain	Residues	Atoms	ZeroOcc	AltConf
6	A	116	Total O<br>116 116	0	0
6	B	120	Total O<br>120 120	0	0
6	C	132	Total O<br>132 132	0	0
6	D	148	Total O<br>148 148	0	0

For Manuscript Review

3 Residue-property plots (i)

These plots are drawn for all protein, RNA, DNA and oligosaccharide chains in the entry. The first graphic for a chain summarises the proportions of the various outlier classes displayed in the second graphic. The second graphic shows the sequence view annotated by issues in geometry and electron density. Residues are color-coded according to the number of geometric quality criteria for which they contain at least one outlier: green = 0, yellow = 1, orange = 2 and red = 3 or more. A red dot above a residue indicates a poor fit to the electron density (RSRZ > 2). Stretches of 2 or more consecutive residues without any outlier are shown as a green connector. Residues present in the sample, but not in the model, are shown in grey.

• Molecule 1: Dihydropteridine reductase

Chain A:

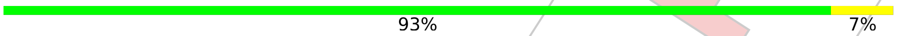

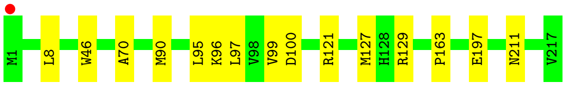

• Molecule 1: Dihydropteridine reductase

Chain B:

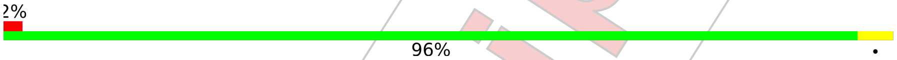

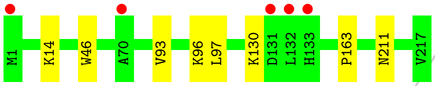

• Molecule 1: Dihydropteridine reductase

Chain C:

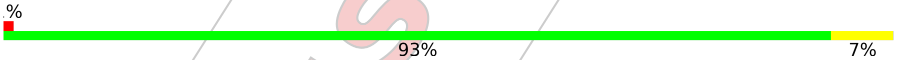

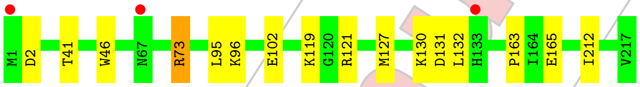

• Molecule 1: Dihydropteridine reductase

Chain D:

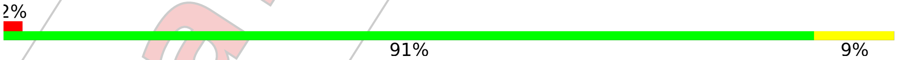

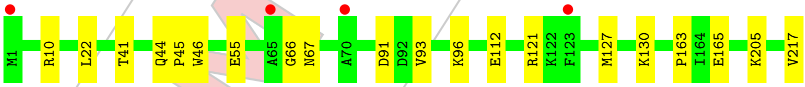

For Manuscript Review

4 Data and refinement statistics (i)

Property	Value	Source
Space group	P 21 21 21	Depositor
Cell constants<br>a, b, c, α, β, γ	87.22Å 95.59Å 112.31Å<br>90.00° 90.00° 90.00°	Depositor
Resolution (Å)	72.79 – 1.98<br>72.79 – 1.98	Depositor<br>EDS
% Data completeness<br>(in resolution range)	100.0 (72.79-1.98)<br>99.9 (72.79-1.98)	Depositor<br>EDS
R_{merge}	0.15	Depositor
R_{sym}	(Not available)	Depositor
$< I/\sigma(I) >$ [1]	2.76 (at 1.98Å)	Xtriage
Refinement program	REFMAC 5.8.0232	Depositor
R, R_{free}	0.171 , 0.219<br>0.182 , 0.225	Depositor<br>DCC
R_{free} test set	3294 reflections (4.99%)	wwPDB-VP
Wilson B-factor (Å^2)	14.7	Xtriage
Anisotropy	0.547	Xtriage
Bulk solvent k_{sol}(e/Å^3), B_{sol}(Å^2)	0.37 , 54.2	EDS
L-test for twinning[2]	$< \|L\| > = 0.49$, $< L^2 > = 0.33$	Xtriage
Estimated twinning fraction	No twinning to report.	Xtriage
F_o,F_c correlation	0.96	EDS
Total number of atoms	7332	wwPDB-VP
Average B, all atoms (Å^2)	18.0	wwPDB-VP

Xtriage's analysis on translational NCS is as follows: *The analyses of the Patterson function reveals a significant off-origin peak that is 42.29 % of the origin peak, indicating pseudo-translational symmetry. The chance of finding a peak of this or larger height randomly in a structure without pseudo-translational symmetry is equal to 2.0850e-04. The detected translational NCS is most likely also responsible for the elevated intensity ratio.*

[1] Intensities estimated from amplitudes.
[2] Theoretical values of $< |L| >$, $< L^2 >$ for acentric reflections are 0.5, 0.333 respectively for untwinned datasets, and 0.375, 0.2 for perfectly twinned datasets.

For Manuscript Review

5 Model quality (i)

5.1 Standard geometry (i)

Bond lengths and bond angles in the following residue types are not validated in this section: FMN, P4K, ACT, CL

The Z score for a bond length (or angle) is the number of standard deviations the observed value is removed from the expected value. A bond length (or angle) with $|Z| > 5$ is considered an outlier worth inspection. RMSZ is the root-mean-square of all Z scores of the bond lengths (or angles).

Mol	Chain	Bond lengths		Bond angles	
		RMSZ	#$\|Z\|$ >5	RMSZ	#$\|Z\|$ >5
1	A	0.74	1/1695 (0.1%)	0.81	0/2298
1	B	0.75	0/1699	0.83	0/2303
1	C	0.74	0/1690	0.82	2/2289 (0.1%)
1	D	0.73	0/1680	0.82	2/2280 (0.1%)
All	All	0.74	1/6764 (0.0%)	0.82	4/9170 (0.0%)

All (1) bond length outliers are listed below:

Mol	Chain	Res	Type	Atoms	Z	Observed(Å)	Ideal(Å)
1	A	70	ALA	C-O	5.11	1.33	1.23

All (4) bond angle outliers are listed below:

Mol	Chain	Res	Type	Atoms	Z	Observed(^{o})	Ideal(^{o})
1	C	73	ARG	NE-CZ-NH1	7.04	123.82	120.30
1	D	217	VAL	CA-C-O	-6.67	106.09	120.10
1	C	73	ARG	NE-CZ-NH2	-5.64	117.48	120.30
1	D	121	ARG	NE-CZ-NH2	-5.11	117.75	120.30

There are no chirality outliers.

There are no planarity outliers.

5.2 Too-close contacts (i)

In the following table, the Non-H and H(model) columns list the number of non-hydrogen atoms and hydrogen atoms in the chain respectively. The H(added) column lists the number of hydrogen atoms added and optimized by MolProbity. The Clashes column lists the number of clashes within the asymmetric unit, whereas Symm-Clashes lists symmetry-related clashes.

For Manuscript Review

Mol	Chain	Non-H	H(model)	H(added)	Clashes	Symm-Clashes
1	A	1665	0	1624	15	0
1	B	1668	0	1632	4	0
1	C	1663	0	1622	15	0
1	D	1656	0	1614	19	0
2	A	31	0	19	2	0
2	B	31	0	19	3	0
2	C	31	0	19	2	0
2	D	31	0	19	2	0
3	A	4	0	3	0	0
3	B	4	0	3	1	0
3	C	16	0	12	6	0
3	D	8	0	6	8	0
4	C	7	0	0	0	0
5	D	1	0	0	1	0
6	A	116	0	0	3	0
6	B	120	0	0	1	1
6	C	132	0	0	1	0
6	D	148	0	0	1	1
All	All	7332	0	6592	53	1

The all-atom clashscore is defined as the number of clashes found per 1000 atoms (including hydrogen atoms). The all-atom clashscore for this structure is 4.

All (53) close contacts within the same asymmetric unit are listed below, sorted by their clash magnitude.

Atom-1	Atom-2	Interatomic distance (Å)	Clash overlap (Å)
1:D:96:LYS:NZ	1:D:96:LYS:CD	2.16	1.08
1:C:130:LYS:NZ	6:C:401:HOH:O	1.97	0.98
1:C:131:ASP:CB	1:C:131:ASP:OD2	2.18	0.92
1:D:112:GLU:OE1	1:D:112:GLU:CB	2.22	0.86
1:C:41:THR:H	3:C:304:ACT:H3	1.40	0.84
1:C:119:LYS:CG	1:C:119:LYS:NZ	2.40	0.84
1:D:41:THR:H	3:D:302:ACT:H3	1.41	0.84
1:D:127:MET:CB	1:D:127:MET:SD	2.66	0.83
1:A:95:LEU:HD22	1:A:121:ARG:HD3	1.65	0.79
3:B:302:ACT:H1	6:B:498:HOH:O	1.87	0.74
1:C:41:THR:N	3:C:304:ACT:H3	2.04	0.72
1:D:41:THR:N	3:D:302:ACT:H3	2.06	0.70
1:A:99:VAL:CG2	6:A:413:HOH:O	2.41	0.68
1:A:127:MET:CB	1:A:127:MET:SD	2.82	0.68
1:C:127:MET:SD	1:C:127:MET:CB	2.82	0.67
1:C:96:LYS:CG	1:C:96:LYS:NZ	2.57	0.66

Continued on next page...

For Manuscript Review

Continued from previous page...

Atom-1	Atom-2	Interatomic distance (Å)	Clash overlap (Å)
1:A:95:LEU:O	1:A:99:VAL:HG23	1.96	0.65
1:D:41:THR:H	3:D:302:ACT:CH3	2.10	0.64
1:C:41:THR:H	3:C:304:ACT:CH3	2.10	0.63
1:A:163:PRO:HG2	2:A:301:FMN:C9	2.28	0.62
1:C:163:PRO:HG2	2:C:301:FMN:C9	2.31	0.61
1:D:41:THR:CA	3:D:302:ACT:H3	2.33	0.59
1:D:41:THR:HA	3:D:302:ACT:H3	1.85	0.59
1:D:10:ARG:HA	5:D:304:CL:CL	2.42	0.57
1:D:112:GLU:OE1	1:D:112:GLU:OE2	2.23	0.56
1:C:41:THR:CA	3:C:304:ACT:H3	2.35	0.56
1:A:99:VAL:HG22	1:A:121:ARG:NH1	2.22	0.55
1:C:41:THR:HA	3:C:304:ACT:H3	1.89	0.55
1:A:99:VAL:HG22	6:A:413:HOH:O	2.07	0.55
1:D:163:PRO:HG2	2:D:301:FMN:C9	2.38	0.54
1:B:163:PRO:HG2	2:B:301:FMN:C9	2.39	0.52
1:D:96:LYS:NZ	1:D:96:LYS:CG	2.73	0.51
1:D:55:GLU:OE2	1:D:55:GLU:OE1	2.29	0.50
1:A:96:LYS:HE2	1:A:100:ASP:OD1	2.13	0.48
1:D:205:LYS:H	3:D:303:ACT:CH3	2.28	0.47
1:A:121:ARG:HD2	6:A:413:HOH:O	2.15	0.47
1:A:90:MET:O	1:A:129:ARG:HD3	2.15	0.46
1:C:95:LEU:HD22	1:C:121:ARG:HD3	1.98	0.46
1:D:22:LEU:O	6:D:401:HOH:O	2.20	0.46
1:A:211:ASN:HD21	1:B:130:LYS:HE3	1.81	0.45
1:D:205:LYS:H	3:D:303:ACT:H1	1.83	0.44
1:A:8:LEU:HD12	1:B:96:LYS:HD3	2.01	0.43
1:C:165:GLU:HG3	2:C:301:FMN:H6	2.00	0.43
2:A:301:FMN:O2'	3:C:304:ACT:H1	2.18	0.43
1:C:102:GLU:OE2	1:C:121:ARG:NH2	2.51	0.43
1:D:91:ASP:OD2	1:D:93:VAL:HG22	2.19	0.43
1:A:97:LEU:HD21	1:C:212:ILE:HD13	2.01	0.42
1:B:14:LYS:NZ	2:B:301:FMN:O2	2.47	0.42
1:D:44:GLN:N	1:D:45:PRO:HD3	2.35	0.42
1:A:99:VAL:HG22	1:A:121:ARG:HH11	1.85	0.41
1:A:197:GLU:OE2	1:A:197:GLU:CB	2.68	0.40
2:B:301:FMN:O2'	3:D:302:ACT:H1	2.20	0.40
1:D:165:GLU:HG3	2:D:301:FMN:H6	2.02	0.40

All (1) symmetry-related close contacts are listed below. The label for Atom-2 includes the symmetry operator and encoded unit-cell translations to be applied.

For Manuscript Review

Atom-1	Atom-2	Interatomic distance (Å)	Clash overlap (Å)
6:B:513:HOH:O	6:D:508:HOH:O[2_565]	2.17	0.03

5.3 Torsion angles (i)

5.3.1 Protein backbone (i)

In the following table, the Percentiles column shows the percent Ramachandran outliers of the chain as a percentile score with respect to all X-ray entries followed by that with respect to entries of similar resolution.

The Analysed column shows the number of residues for which the backbone conformation was analysed, and the total number of residues.

Mol	Chain	Analysed	Favoured	Allowed	Outliers	Percentiles	
1	A	215/217 (99%)	214 (100%)	1 (0%)	0	100	100
1	B	215/217 (99%)	211 (98%)	4 (2%)	0	100	100
1	C	215/217 (99%)	210 (98%)	5 (2%)	0	100	100
1	D	215/217 (99%)	210 (98%)	3 (1%)	2 (1%)	17	8
All	All	860/868 (99%)	845 (98%)	13 (2%)	2 (0%)	47	38

All (2) Ramachandran outliers are listed below:

Mol	Chain	Res	Type
1	D	66	GLY
1	D	67	ASN

5.3.2 Protein sidechains (i)

In the following table, the Percentiles column shows the percent sidechain outliers of the chain as a percentile score with respect to all X-ray entries followed by that with respect to entries of similar resolution.

The Analysed column shows the number of residues for which the sidechain conformation was analysed, and the total number of residues.

Mol	Chain	Analysed	Rotameric	Outliers	Percentiles	
1	A	170/178 (96%)	169 (99%)	1 (1%)	86	85
1	B	172/178 (97%)	168 (98%)	4 (2%)	50	44
1	C	170/178 (96%)	166 (98%)	4 (2%)	49	41

Continued on next page...

For Manuscript Review

Continued from previous page...

Mol	Chain	Analysed	Rotameric	Outliers	Percentiles	
1	D	168/178 (94%)	166 (99%)	2 (1%)	71	67
All	All	680/712 (96%)	669 (98%)	11 (2%)	62	56

All (11) residues with a non-rotameric sidechain are listed below:

Mol	Chain	Res	Type
1	A	46	TRP
1	B	46	TRP
1	B	93	VAL
1	B	97	LEU
1	B	211	ASN
1	C	2	ASP
1	C	46	TRP
1	C	73	ARG
1	C	132	LEU
1	D	46	TRP
1	D	130	LYS

Sometimes sidechains can be flipped to improve hydrogen bonding and reduce clashes. All (2) such sidechains are listed below:

Mol	Chain	Res	Type
1	A	211	ASN
1	B	117	ASN

5.3.3 RNA (i)

There are no RNA molecules in this entry.

5.4 Non-standard residues in protein, DNA, RNA chains (i)

There are no non-standard protein/DNA/RNA residues in this entry.

5.5 Carbohydrates (i)

There are no monosaccharides in this entry.

For Manuscript Review

5.6 Ligand geometry (i)

Of 14 ligands modelled in this entry, 1 is monoatomic - leaving 13 for Mogul analysis.

In the following table, the Counts columns list the number of bonds (or angles) for which Mogul statistics could be retrieved, the number of bonds (or angles) that are observed in the model and the number of bonds (or angles) that are defined in the Chemical Component Dictionary. The Link column lists molecule types, if any, to which the group is linked. The Z score for a bond length (or angle) is the number of standard deviations the observed value is removed from the expected value. A bond length (or angle) with $|Z| > 2$ is considered an outlier worth inspection. RMSZ is the root-mean-square of all Z scores of the bond lengths (or angles).

Mol	Type	Chain	Res	Link	Bond lengths			Bond angles		
					Counts	RMSZ	#$\|Z\| > 2$	Counts	RMSZ	#$\|Z\| > 2$
3	ACT	A	302	-	3,3,3	0.71	0	3,3,3	0.76	0
3	ACT	C	304	-	3,3,3	1.15	0	3,3,3	0.48	0
2	FMN	B	301	-	33,33,33	1.34	2 (6%)	48,50,50	1.28	7 (14%)
2	FMN	C	301	-	33,33,33	1.41	6 (18%)	48,50,50	1.38	6 (12%)
3	ACT	B	302	-	3,3,3	1.31	0	3,3,3	1.00	0
3	ACT	D	303	-	3,3,3	0.98	0	3,3,3	0.63	0
2	FMN	D	301	-	33,33,33	1.58	6 (18%)	48,50,50	1.70	11 (22%)
3	ACT	C	303	-	3,3,3	1.00	0	3,3,3	0.76	0
4	P4K	C	306	-	6,6,44	0.47	0	5,5,43	0.51	0
3	ACT	D	302	-	3,3,3	1.22	0	3,3,3	0.76	0
3	ACT	C	305	-	3,3,3	1.12	0	3,3,3	0.73	0
2	FMN	A	301	-	33,33,33	1.53	5 (15%)	48,50,50	1.43	8 (16%)
3	ACT	C	302	-	3,3,3	1.07	0	3,3,3	0.64	0

In the following table, the Chirals column lists the number of chiral outliers, the number of chiral centers analysed, the number of these observed in the model and the number defined in the Chemical Component Dictionary. Similar counts are reported in the Torsion and Rings columns. '-' means no outliers of that kind were identified.

Mol	Type	Chain	Res	Link	Chirals	Torsions	Rings
2	FMN	B	301	-	-	3/18/18/18	0/3/3/3
2	FMN	C	301	-	-	1/18/18/18	0/3/3/3
2	FMN	D	301	-	-	1/18/18/18	0/3/3/3
4	P4K	C	306	-	-	2/4/4/42	-
2	FMN	A	301	-	-	1/18/18/18	0/3/3/3

All (19) bond length outliers are listed below:

Mol	Chain	Res	Type	Atoms	Z	Observed(Å)	Ideal(Å)
2	D	301	FMN	C9A-C5A	5.40	1.50	1.41

Continued on next page...

For Manuscript Review

Continued from previous page...

Mol	Chain	Res	Type	Atoms	Z	Observed(Å)	Ideal(Å)
2	A	301	FMN	C9A-C5A	4.59	1.48	1.41
2	C	301	FMN	C9A-C5A	4.43	1.48	1.41
2	B	301	FMN	C9A-C5A	4.41	1.48	1.41
2	A	301	FMN	C1'-C2'	3.60	1.57	1.52
2	D	301	FMN	C1'-C2'	3.06	1.57	1.52
2	A	301	FMN	C4A-N5	2.76	1.36	1.30
2	C	301	FMN	C4-N3	-2.69	1.33	1.38
2	D	301	FMN	C4-N3	-2.42	1.34	1.38
2	A	301	FMN	C8-C7	2.30	1.46	1.40
2	C	301	FMN	C4A-C10	2.29	1.50	1.44
2	C	301	FMN	C1'-C2'	2.27	1.55	1.52
2	C	301	FMN	C8-C7	2.18	1.46	1.40
2	D	301	FMN	O4'-C4'	2.13	1.47	1.43
2	B	301	FMN	C1'-C2'	2.11	1.55	1.52
2	C	301	FMN	C4A-N5	2.08	1.34	1.30
2	D	301	FMN	C4A-N5	2.04	1.34	1.30
2	D	301	FMN	C8-C7	2.03	1.46	1.40
2	A	301	FMN	O2'-C2'	2.03	1.47	1.43

All (32) bond angle outliers are listed below:

Mol	Chain	Res	Type	Atoms	Z	Observed(°)	Ideal(°)
2	D	301	FMN	O2-C2-N1	-4.30	114.70	121.83
2	C	301	FMN	O2'-C2'-C1'	4.00	119.48	109.80
2	D	301	FMN	O2'-C2'-C1'	3.76	118.89	109.80
2	D	301	FMN	N3-C2-N1	3.61	126.46	119.38
2	A	301	FMN	O2-C2-N1	-3.36	116.25	121.83
2	C	301	FMN	O2-C2-N1	-3.23	116.47	121.83
2	B	301	FMN	C4A-C10-N10	3.09	120.99	116.48
2	A	301	FMN	O4-C4-C4A	-3.07	118.46	126.60
2	A	301	FMN	C4-C4A-N5	3.02	122.52	118.23
2	D	301	FMN	C5A-C9A-N10	2.98	121.03	117.95
2	D	301	FMN	C9A-C5A-N5	-2.87	119.31	122.43
2	D	301	FMN	C4A-C10-N10	2.81	120.59	116.48
2	C	301	FMN	C5A-C9A-N10	2.80	120.85	117.95
2	B	301	FMN	O2-C2-N1	-2.73	117.31	121.83
2	D	301	FMN	C4-N3-C2	-2.64	120.77	125.64
2	D	301	FMN	O4-C4-C4A	-2.57	119.77	126.60
2	D	301	FMN	P-O5'-C5'	2.52	125.22	118.30
2	A	301	FMN	O2'-C2'-C1'	2.47	115.78	109.80
2	B	301	FMN	O2'-C2'-C1'	2.45	115.73	109.80
2	C	301	FMN	N3-C2-N1	2.43	124.16	119.38

Continued on next page...

For Manuscript Review

Continued from previous page...

Mol	Chain	Res	Type	Atoms	Z	Observed(°)	Ideal(°)
2	B	301	FMN	C4-C4A-N5	2.32	121.53	118.23
2	B	301	FMN	C1'-N10-C9A	2.20	124.19	120.51
2	B	301	FMN	C9A-N10-C10	-2.19	117.36	120.77
2	C	301	FMN	C9A-N10-C10	-2.17	117.38	120.77
2	D	301	FMN	C9A-N10-C10	-2.15	117.42	120.77
2	D	301	FMN	O5'-P-O1P	-2.11	100.56	106.47
2	A	301	FMN	C4A-C4-N3	2.10	118.52	113.19
2	B	301	FMN	C4A-C10-N1	-2.08	119.90	124.73
2	A	301	FMN	C4'-C3'-C2'	2.05	117.62	113.36
2	A	301	FMN	O5'-P-O1P	-2.03	100.78	106.47
2	A	301	FMN	N3-C2-N1	2.02	123.35	119.38
2	C	301	FMN	O5'-P-O1P	-2.00	100.85	106.47

There are no chirality outliers.

All (8) torsion outliers are listed below:

Mol	Chain	Res	Type	Atoms
4	C	306	P4K	O1-C1-C2-O2
4	C	306	P4K	C4-C3-O2-C2
2	D	301	FMN	C4'-C5'-O5'-P
2	C	301	FMN	C4'-C5'-O5'-P
2	A	301	FMN	C4'-C5'-O5'-P
2	B	301	FMN	C4'-C5'-O5'-P
2	B	301	FMN	N10-C1'-C2'-O2'
2	B	301	FMN	C2'-C1'-N10-C10

There are no ring outliers.

8 monomers are involved in 22 short contacts:

Mol	Chain	Res	Type	Clashes	Symm-Clashes
3	C	304	ACT	6	0
2	B	301	FMN	3	0
2	C	301	FMN	2	0
3	B	302	ACT	1	0
3	D	303	ACT	2	0
2	D	301	FMN	2	0
3	D	302	ACT	6	0
2	A	301	FMN	2	0

The following is a two-dimensional graphical depiction of Mogul quality analysis of bond lengths, bond angles, torsion angles, and ring geometry for all instances of the Ligand of Interest. In

For Manuscript Review

addition, ligands with molecular weight > 250 and outliers as shown on the validation Tables will also be included. For torsion angles, if less then 5% of the Mogul distribution of torsion angles is within 10 degrees of the torsion angle in question, then that torsion angle is considered an outlier. Any bond that is central to one or more torsion angles identified as an outlier by Mogul will be highlighted in the graph. For rings, the root-mean-square deviation (RMSD) between the ring in question and similar rings identified by Mogul is calculated over all ring torsion angles. If the average RMSD is greater than 60 degrees and the minimal RMSD between the ring in question and any Mogul-identified rings is also greater than 60 degrees, then that ring is considered an outlier. The outliers are highlighted in purple. The color gray indicates Mogul did not find sufficient equivalents in the CSD to analyse the geometry.

**Ligand FMN B 301**	
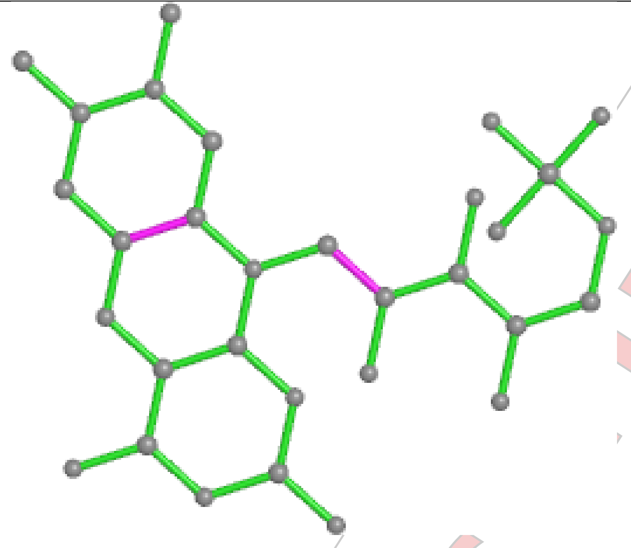 **Bond lengths**	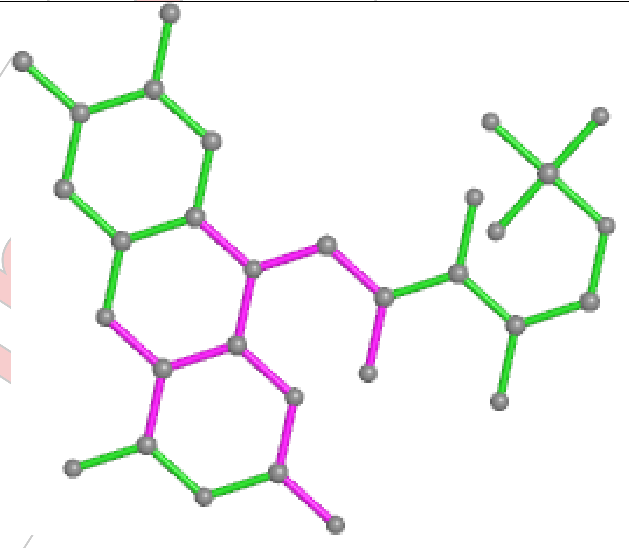 **Bond angles**
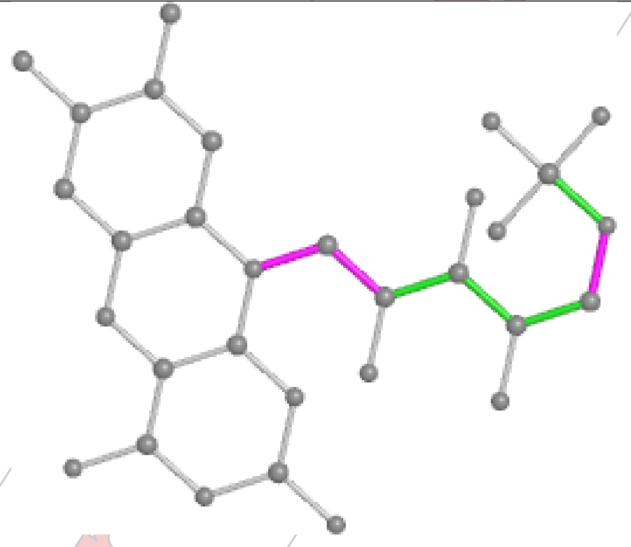 **Torsions**	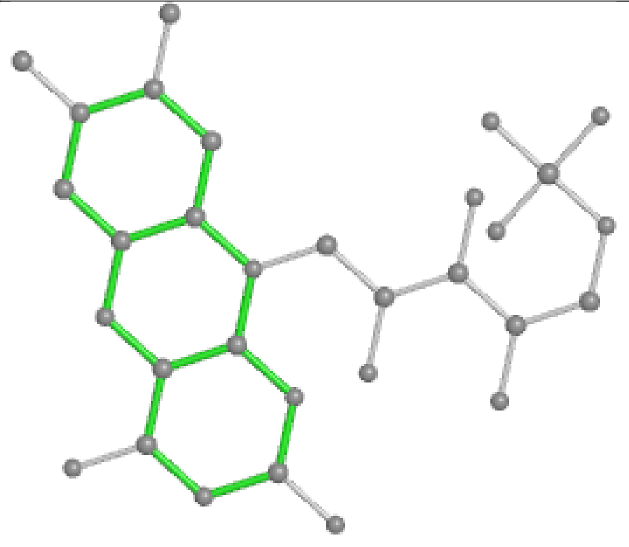 **Rings**

For Review

Ligand FMN C 301

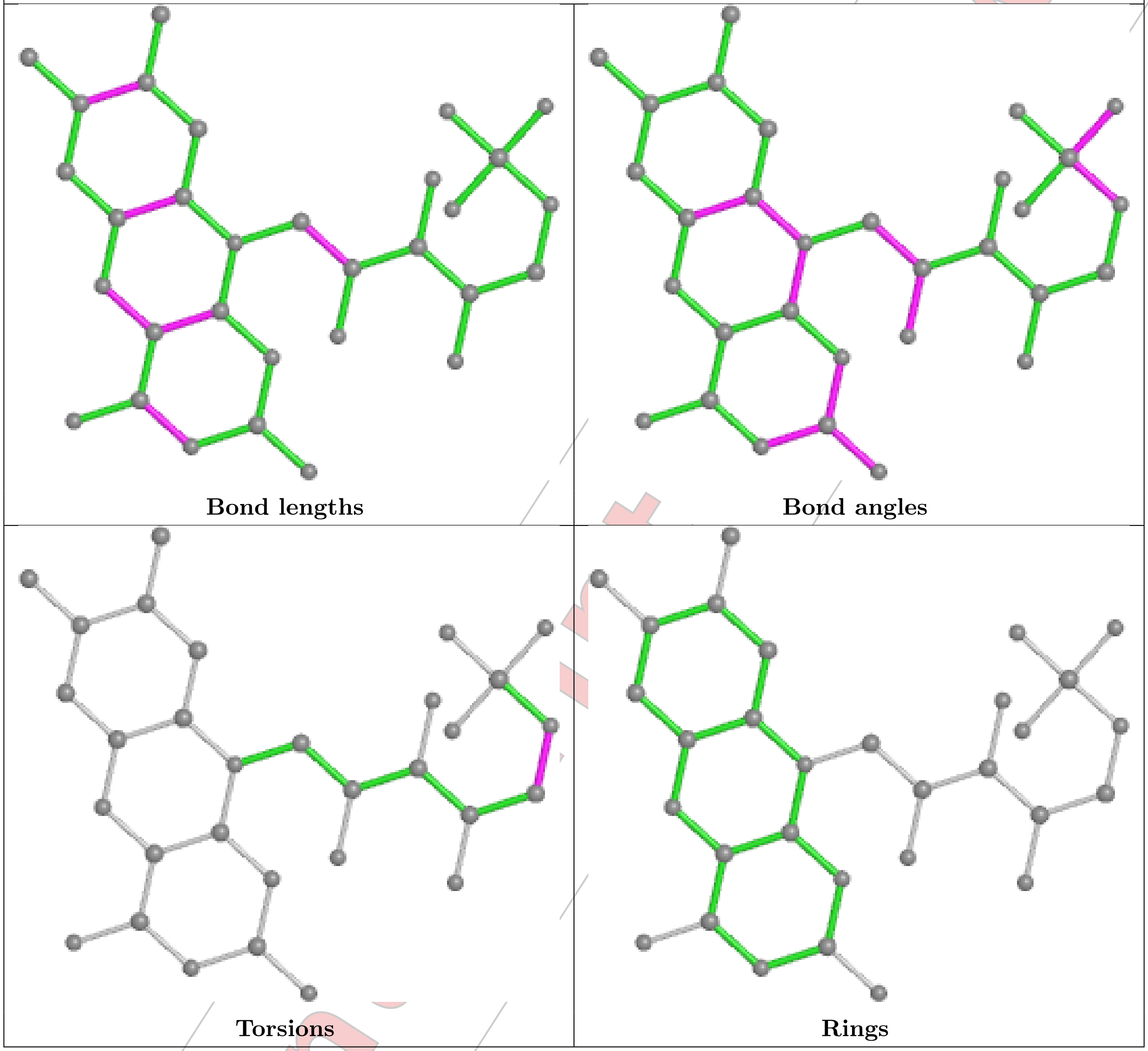

For Manuscript Review

Ligand FMN D 301

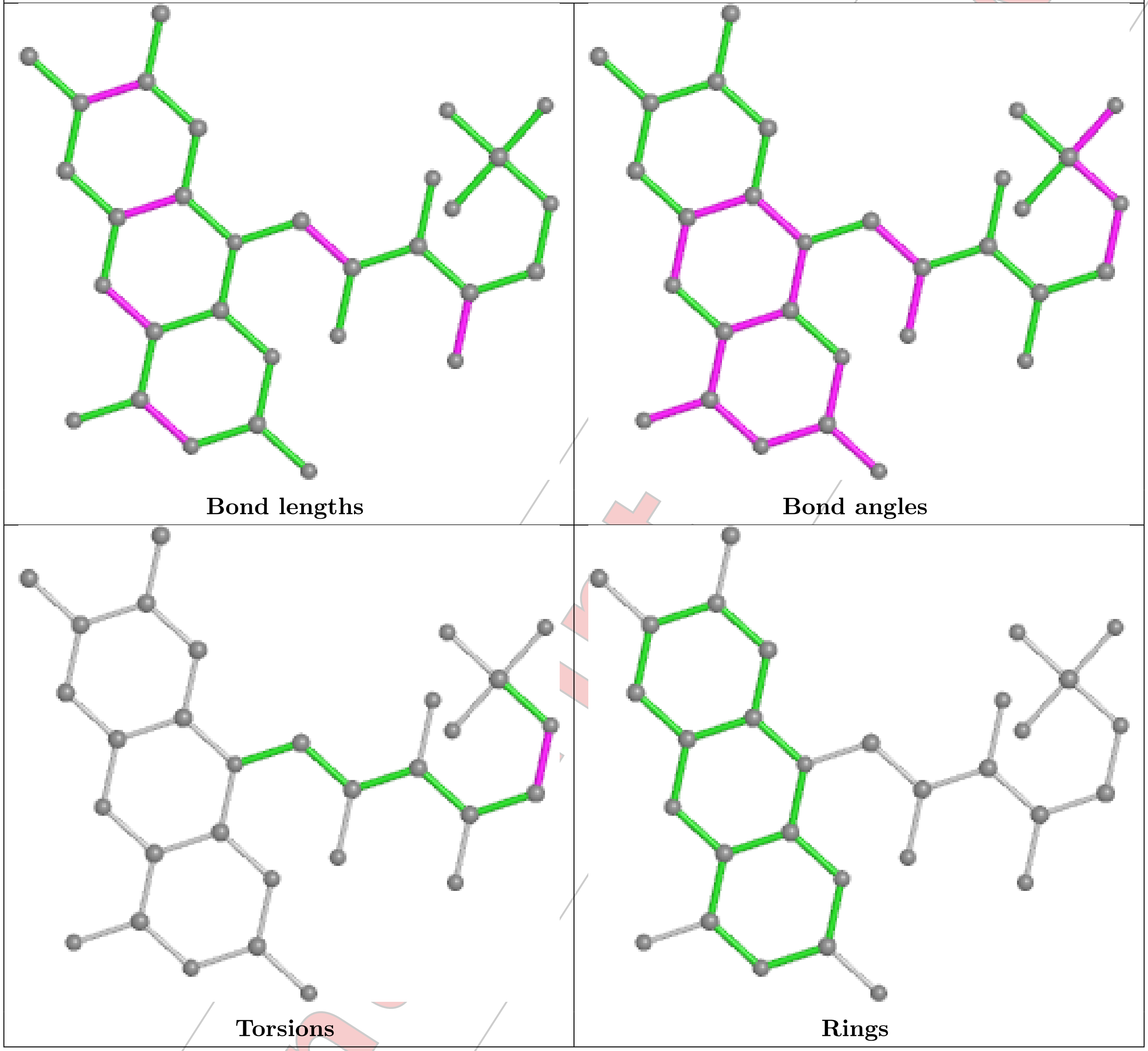

Ligand P4K C 306

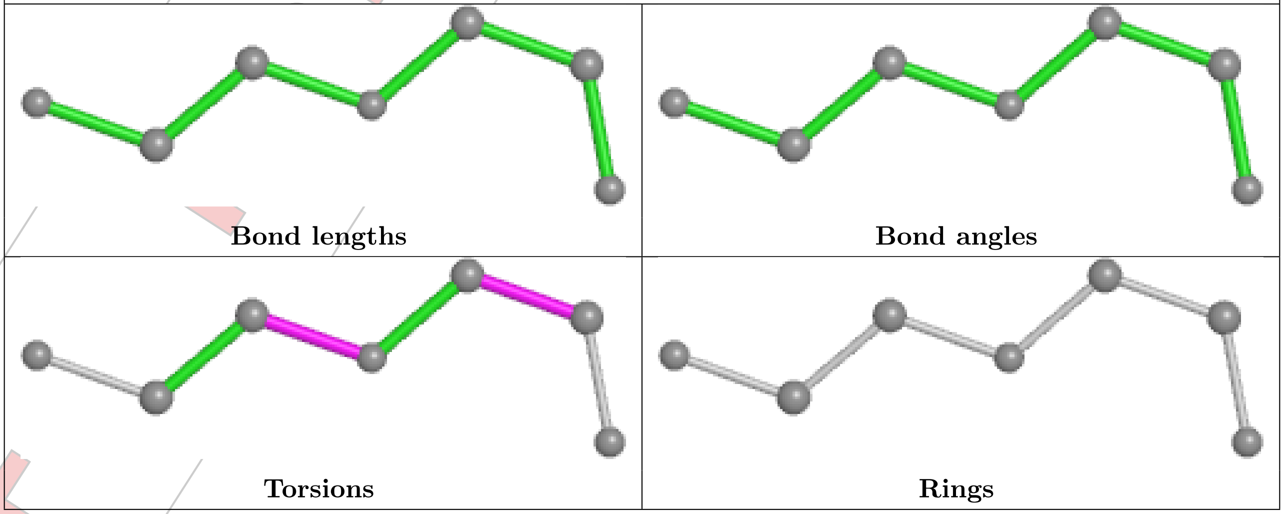

Ligand FMN A 301

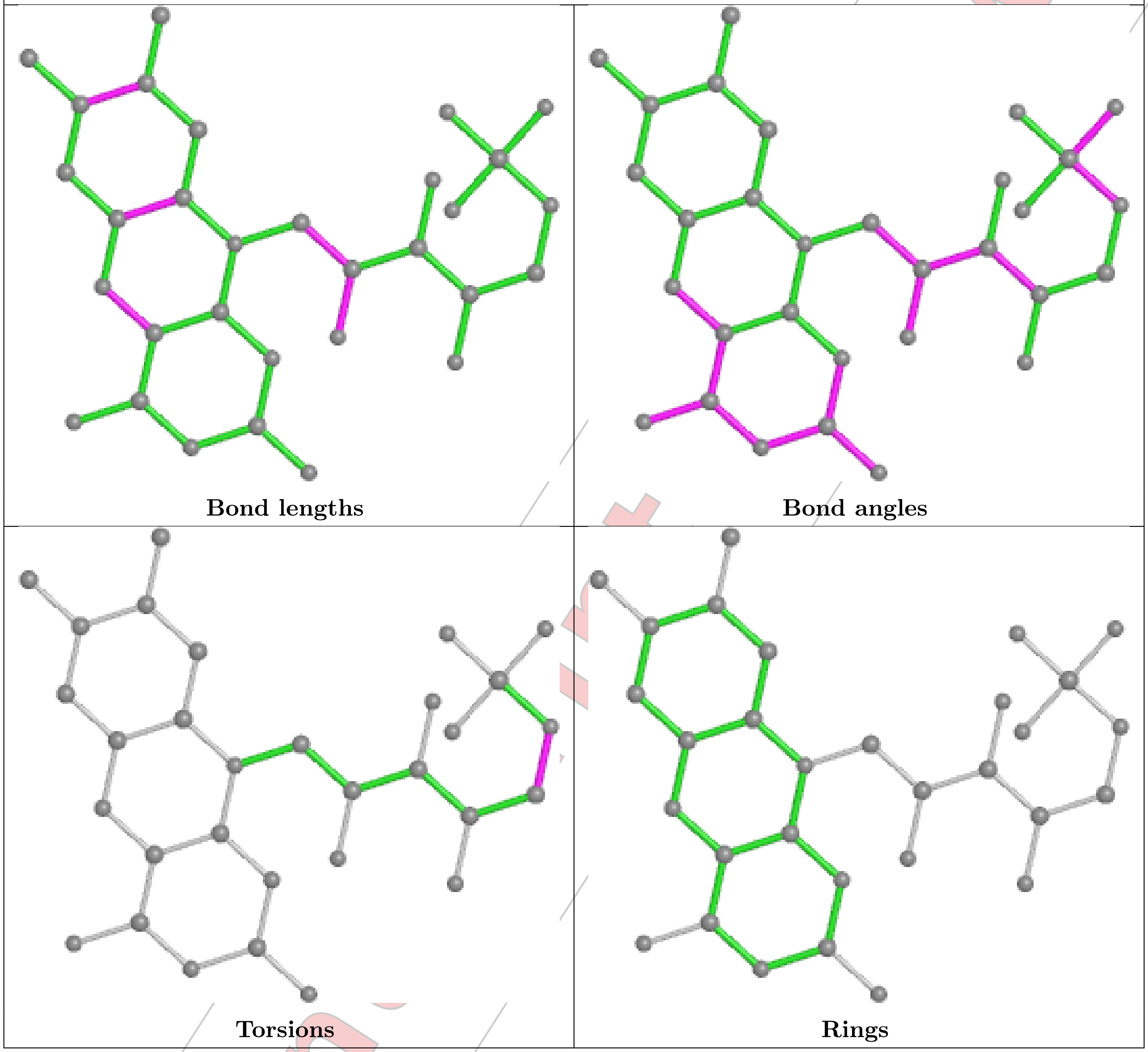

5.7 Other polymers (i)

There are no such residues in this entry.

5.8 Polymer linkage issues (i)

There are no chain breaks in this entry.

For Manuscript Review

6 Fit of model and data (i)

6.1 Protein, DNA and RNA chains (i)

In the following table, the column labelled '#RSRZ> 2' contains the number (and percentage) of RSRZ outliers, followed by percent RSRZ outliers for the chain as percentile scores relative to all X-ray entries and entries of similar resolution. The OWAB column contains the minimum, median, 95th percentile and maximum values of the occupancy-weighted average B-factor per residue. The column labelled 'Q< 0.9' lists the number of (and percentage) of residues with an average occupancy less than 0.9.

Mol	Chain	Analysed	<RSRZ>	#RSRZ>2			OWAB(Å^2)	Q<0.9
1	A	217/217 (100%)	-0.19	1 (0%)	91	91	7, 15, 34, 47	1 (0%)
1	B	217/217 (100%)	-0.12	5 (2%)	60	62	7, 15, 35, 62	0
1	C	217/217 (100%)	-0.24	3 (1%)	75	77	7, 14, 32, 68	0
1	D	217/217 (100%)	-0.21	4 (1%)	68	69	7, 15, 34, 61	0
All	All	868/868 (100%)	-0.19	13 (1%)	73	75	7, 15, 34, 68	1 (0%)

All (13) RSRZ outliers are listed below:

Mol	Chain	Res	Type	RSRZ
1	C	1	MET	5.6
1	D	1	MET	3.6
1	B	1	MET	3.2
1	B	133	HIS	3.2
1	A	1	MET	3.0
1	D	123	PHE	2.9
1	D	65	ALA	2.6
1	B	132	LEU	2.5
1	B	131	ASP	2.4
1	C	133	HIS	2.4
1	C	67	ASN	2.3
1	B	70	ALA	2.2
1	D	70	ALA	2.0

6.2 Non-standard residues in protein, DNA, RNA chains (i)

There are no non-standard protein/DNA/RNA residues in this entry.

For Manuscript Review

6.3 Carbohydrates (i)

There are no monosaccharides in this entry.

6.4 Ligands (i)

In the following table, the Atoms column lists the number of modelled atoms in the group and the number defined in the chemical component dictionary. The B-factors column lists the minimum, median, 95th percentile and maximum values of B factors of atoms in the group. The column labelled 'Q< 0.9' lists the number of atoms with occupancy less than 0.9.

Mol	Type	Chain	Res	Atoms	RSCC	RSR	B-factors(Å^2)	Q<0.9
3	ACT	D	302	4/4	0.74	0.25	18,24,29,33	0
3	ACT	C	304	4/4	0.75	0.24	17,24,27,31	0
3	ACT	D	303	4/4	0.84	0.14	32,38,40,43	0
3	ACT	C	305	4/4	0.88	0.17	38,40,41,41	0
4	P4K	C	306	7/45	0.88	0.23	20,34,42,47	0
3	ACT	C	303	4/4	0.90	0.21	51,52,53,59	0
3	ACT	B	302	4/4	0.92	0.23	17,26,28,31	0
3	ACT	A	302	4/4	0.93	0.19	23,34,34,43	0
3	ACT	C	302	4/4	0.94	0.19	33,33,35,40	0
5	CL	D	304	1/1	0.95	0.28	63,63,63,63	0
2	FMN	A	301	31/31	0.96	0.11	11,14,16,16	0
2	FMN	C	301	31/31	0.97	0.10	9,11,13,14	0
2	FMN	D	301	31/31	0.97	0.09	7,10,11,14	0
2	FMN	B	301	31/31	0.97	0.11	10,11,13,14	0

The following is a graphical depiction of the model fit to experimental electron density of all instances of the Ligand of Interest. In addition, ligands with molecular weight > 250 and outliers as shown on the geometry validation Tables will also be included. Each fit is shown from different orientation to approximate a three-dimensional view.

For Manuscript Review

Electron density around P4K C 306:

$2mF_o$-DF_c (at 0.7 rmsd) in gray
mF_o-DF_c (at 3 rmsd) in purple (negative)
and green (positive)

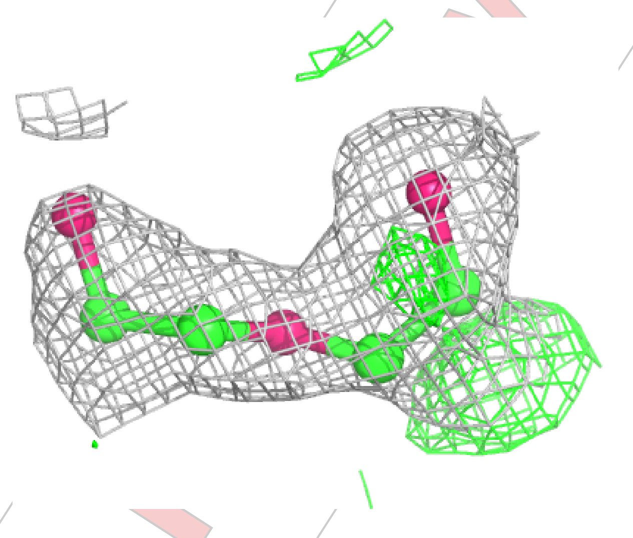

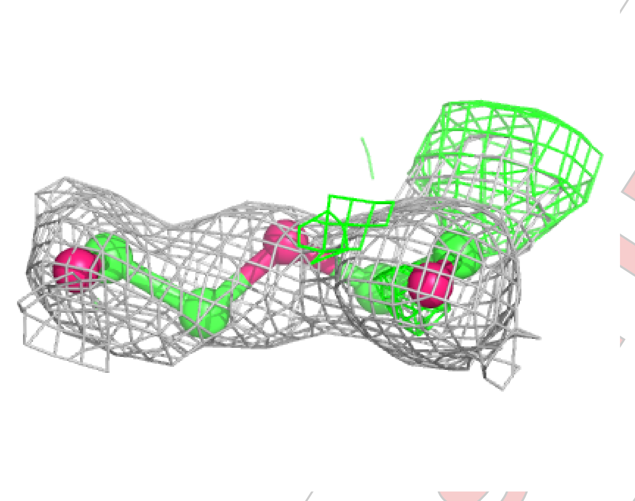

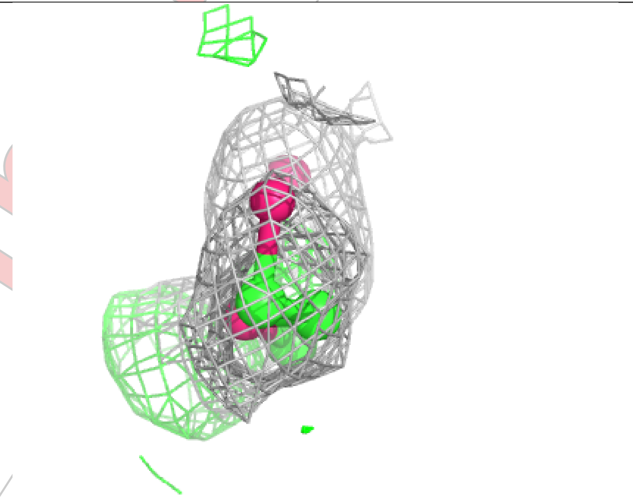

For Manuscript Review

Electron density around FMN A 301:

$2mF_o$-DF_c (at 0.7 rmsd) in gray
mF_o-DF_c (at 3 rmsd) in purple (negative)
and green (positive)

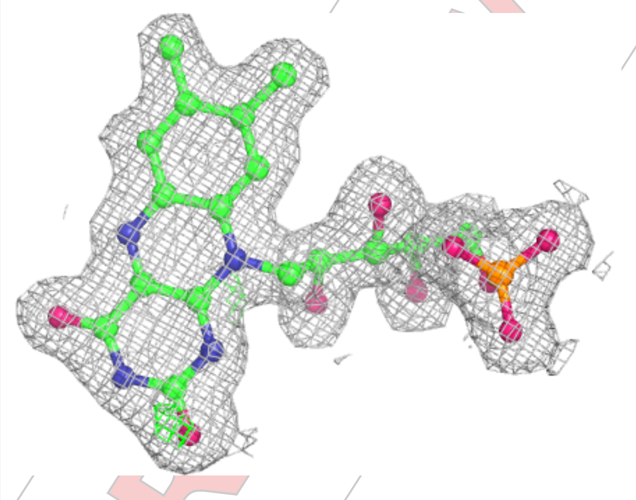

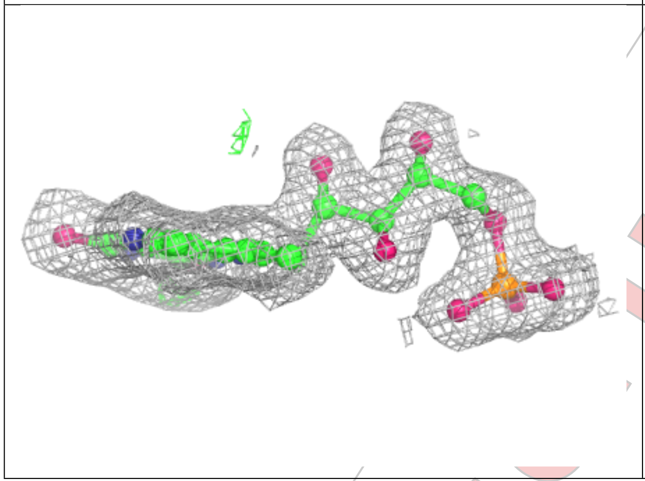

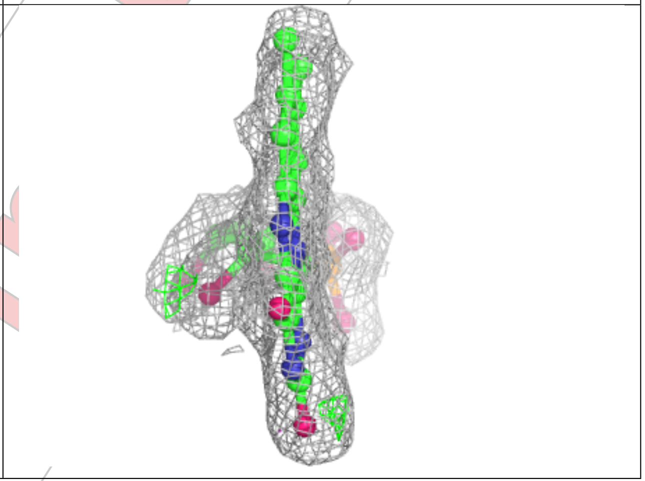

For Manuscript Review

Electron density around FMN C 301:

$2mF_o$-DF_c (at 0.7 rmsd) in gray
mF_o-DF_c (at 3 rmsd) in purple (negative)
and green (positive)

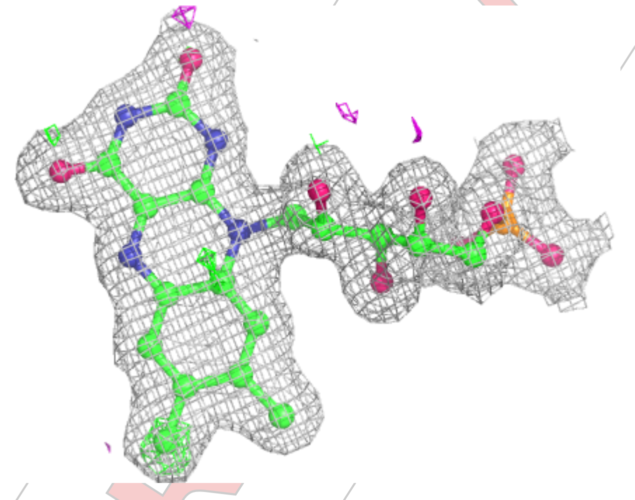

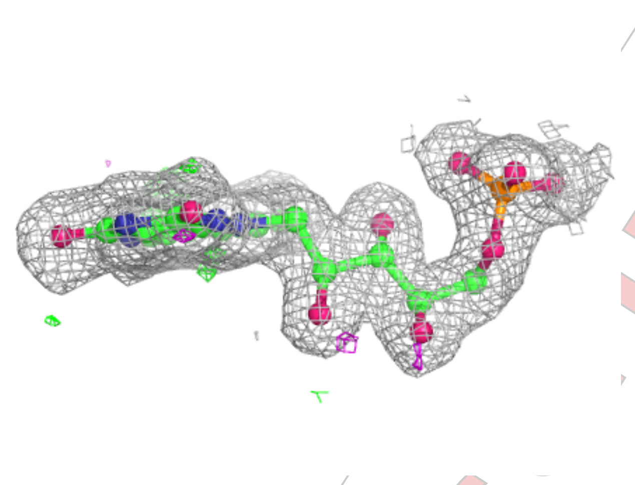

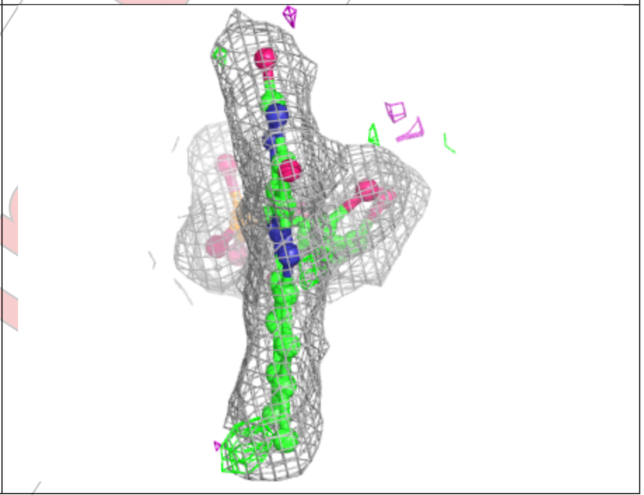

For Manuscript Review

Electron density around FMN D 301:

$2mF_o$-DF_c (at 0.7 rmsd) in gray
mF_o-DF_c (at 3 rmsd) in purple (negative) and green (positive)

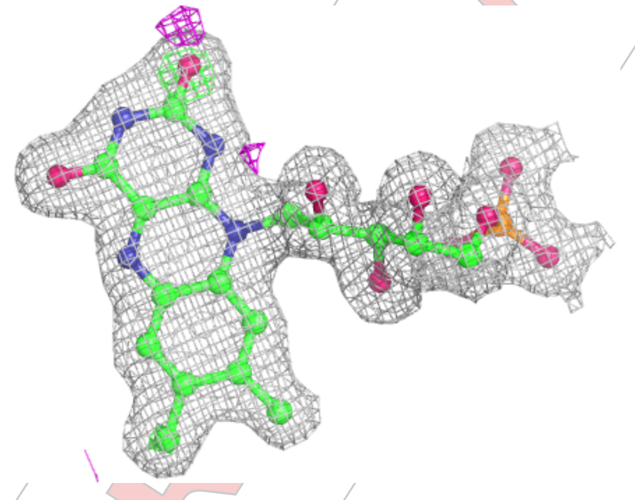

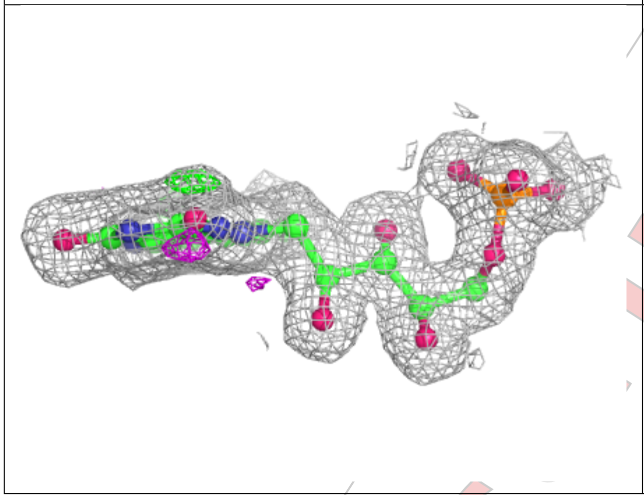

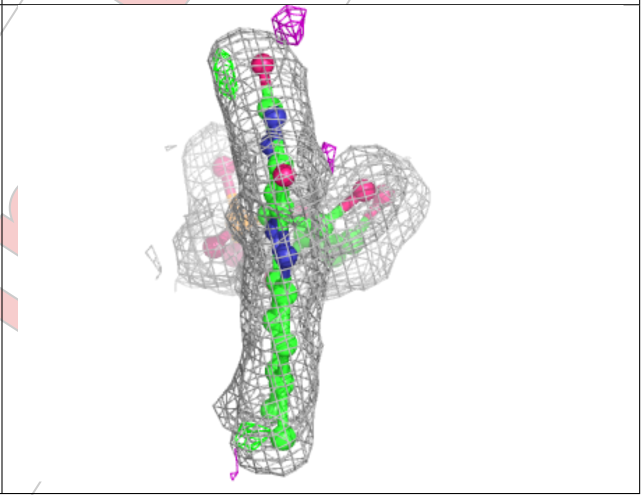

For Manuscript Review

Electron density around FMN B 301:

$2mF_o$-DF_c (at 0.7 rmsd) in gray
mF_o-DF_c (at 3 rmsd) in purple (negative)
and green (positive)

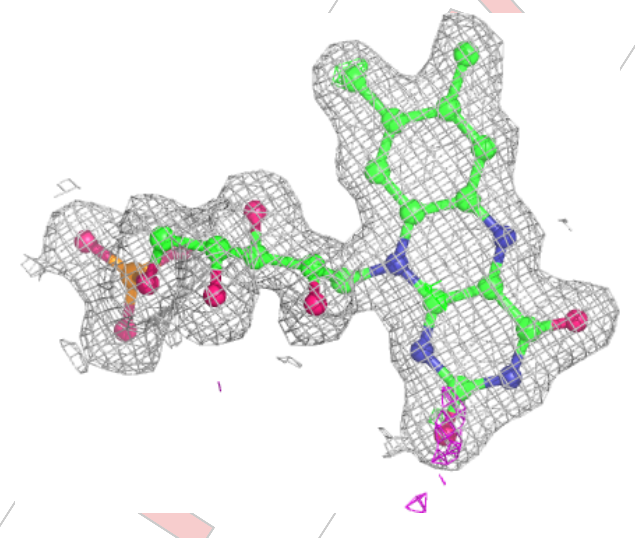

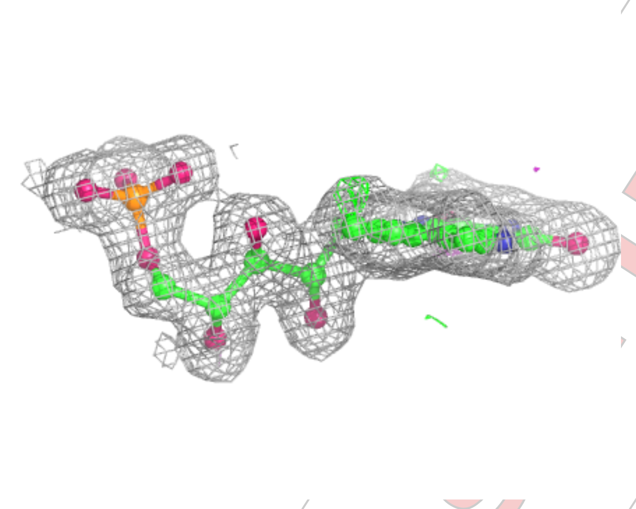

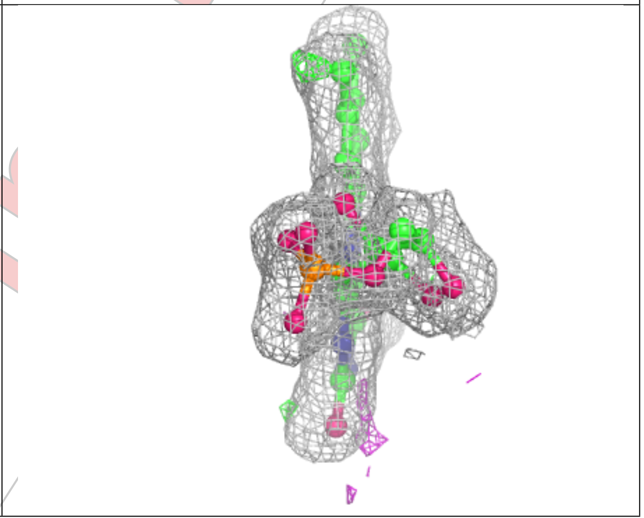

6.5 Other polymers (i)

There are no such residues in this entry.

For Manuscript Review
